# Meat production traits of Lalahan sheep: fattening, slaughter, and carcass characteristics of lambs

**DOI:** 10.5194/aab-69-431-2026

**Published:** 2026-08-19

**Authors:** Rabia Arslan, Necmettin Ünal, Akın Yakan

**Affiliations:** 1 Ankara University, Institute Health Science, Department of Animal Breeding and Husbandry, 06110, Ankara, Türkiye; 2 Ankara University, Faculty of Veterinary Medicine, Department of Animal Breeding and Husbandry, 06110, Ankara, Türkiye

## Abstract

This study investigated the fattening performance, slaughter, and carcass characteristics of Lalahan-genotype lambs at different slaughter weights. The animal material of the study consisted of 36 male Lalahan-genotype lambs, randomly divided into three groups and fed intensively. Six lambs were slaughtered from each group, with average weights of 35 (Gr35), 40 (Gr40), and 45 kg (Gr45). The average daily weight gain (DWG) in each group was 241.50 ± 17.90, 243.33 ± 29.68, and 235.45 ± 26.15 g, respectively, while feed conversion rate (FCR) was 3.590, 3.958, and 4.392, respectively. Cold-dressing percentages were 45.26 ± 0.57 %, 44.66 ± 1.15 %, and 46.11 ± 0.85 %, respectively. Carcass lean and fat percentages differed significantly at 61.82 ± 0.60 %, 61.04 ± 0.47 %, and 57.53 ± 0.74 % ( P < 0.001) and 13.57 ± 0.24 %, 14.05 ± 0.17 %, and 19.46 ± 0.68 % ( P < 0.001). Leg, back, and loin percentages differed significantly at 31.57 ± 0.71 %, 30.04 ± 0.58 %, and 28.41 ± 0.27 % ( P < 0.01); 13.39 ± 0.23 %, 12.63 ± 0.46 %, and 14.72 ± 0.56 % ( P < 0.05); and 8.41 ± 0.13 %, 7.88 ± 0.26 %, and 7.19 ± 0.12 %, respectively ( P < 0.001). The lean / fat ratios in Gr35 and Gr40 were significantly higher than in Gr45 ( P < 0.001), but the meat / bone ratio was similar in all groups. The findings indicate that the Lalahan genotype is similar to or better than the Kıvırcık breed from which its genotype originates in terms of fattening performance, slaughter, and carcass characteristics and has potential for meat production in steppe climatic conditions. Parameters such as average daily weight gain, meat percentage, and fat percentage were evaluated based on a slaughter weight of 40 kg being appropriate.

## 1 Introduction

In sheep breeding, income can be obtained from various yields, although the most important source is lamb meat production. To maximize the production of high-quality lamb meat, it is necessary to raise sheep to slaughter weight in a short time, with a large number of lambs showing a high survival rate and superior fattening performance, carcass, and meat quality characteristics. While increasing population and nutrient demand have increased the demand for red meat with high nutritional value (Eser et al., 2025; Ünal et al., 2006b), in Türkiye, sheep meat only accounts for 9.1 % of red-meat production (TÜİK, 2024). To increase this percentage, it is very important to develop genotypes suitable for Türkiye's particular climatic and pasture conditions and appropriate for lamb production. One method to improve the genotype is crossbreeding, which makes it possible to obtain new genotypes adapted to specific environmental conditions, to make domestic breeds more productive, and to improve specific traits (Akçapınar and Özbeyaz, 2021; Uğurlu et al., 2025; Yakan et al., 2018).

The Lalahan sheep (Kıvırcık × Akkaraman G1) is a genotype developed as part of projects in Türkiye to obtain new genotypes suitable for steppe-region lamb production conditions. The Lalahan sheep has a deep and wide chest, while its waist and rump are flat and wide, and the rump is inclined downwards. Although the back is slightly lower than the body and waist, the body forms a straight line when viewed from the side. The tail has a small layer of fat at the base, while the fat-free part ends at the tarsal joint. It is well adapted to steppe environmental conditions, has good herding instinct, and is easy to manage. Erol (2013) reported estrus, parturition, and twin birth percentages of 98.70 %, 91.56 %, and 25.18 %, respectively, while the average body weight, body length, shoulder height, and chest girth at the end of shearing were 52.29 kg, 67.43 cm, 65.91 cm, and 90.65 cm, respectively. Lalahan sheep exhibit adequate fertility performance and are similar in terms of both genotype and morphology to Kıvırcık lambs. From their study of Kıvırcık lambs fattened to 16.8 kg body weight and slaughtered at 34.70 kg, Altın et al. (2005) reported daily weight gain (DWG) of 250 g and a feed conversion rate (FCR) of 5.30 kg. In the carcass, the arm, shoulder–back–lumbar, rump, neck, and tail percentages were 18.53 %, 22.53 %, 30.59 %, 9.01 %, and 2.58 %, respectively. Akçapınar et al. (2004) and Unal et al. (2006a) determined the milk yield characteristics of Lalahan ewes in the first years of crossbreeding, in a single lactation, and at a single slaughter weight. However, these studies provided no information on fattening performance, slaughter, and carcass characteristics at different slaughter weights. While many studies (Akçapınar et al., 2004; Altın et al., 2005; Yalcintan et al., 2017; Yılmaz et al., 2009) have examined the fattening performance, carcass, and meat quality characteristics of Kıvırcık and Akkaraman breeds, from which the Lalahan genotype is derived, no studies have examined these issues in Lalahan sheep. Thus, to the best of our knowledge, the present study is the first to investigate the fattening, slaughter, and carcass traits at different slaughter weights in Lalahan lambs.

## 2 Material and methods

### 2.1 Animals and feeding

The lambs used in the trial were born as singletons during the same lambing season and were raised under the same herd management practices from weaning until slaughter. Care was taken to ensure that the ewes had given birth at least twice. During the pre-weaning period, all lambs were raised alongside their mothers under similar care and feeding conditions, and routine herd management practices were followed. Thirty-six healthy male Lalahan lambs, weaned at approximately 90 d of age, were selected for the experiment and subsequently randomly assigned to the experimental groups. Therefore, it was assumed that the animals used in the experiment represented the Lalahan genotype within the research flock. The lambs were randomly divided into three groups and fed intensively until reaching an average body weight of 35 kg (Gr35), 40 kg (Gr40), and 45 kg (Gr45) in their respective groups. Fattening was started with 400 g per head d^−1^ of concentrate feed and 300 g per head d^−1^ of alfalfa hay. During fattening, lambs were individually fed ad libitum with concentrate feed and 300 g per head d^−1^ of limited alfalfa hay. The feeds were weighed every morning and evening, and the remaining concentrate and alfalfa hay in the feeders were collected and weighed every day in the morning before feeding and deducted from the total feed consumption. During fattening, each lamb was provided with 2.0 m^2^ of space, 50 cm feeder length, and ad libitum drinking-water access. Table 1 presents the concentrate feed and alfalfa hay nutrient compositions.

**Table 1:** Nutrient composition and energy levels of concentrate feed and alfalfa hay.

Composition	Concentrate	Alfalfa
	feed	hay
Dry matter (%)	90.87	91.96
Crude protein (%)	16.42	13.72
Crude cellulose (%)	11.81	38.74 (ADF)
Crude oil (%)	1.68	1.76
Crude ash (%)	6.78	12.71
Metabolic energy	2700	2080
(kcal kg DM^−1^)		

ADF: acid detergent fiber.

### 2.2 Fattening performance

The amounts of concentrate and alfalfa hay consumed daily in each group during fattening were recorded, while the lambs' body weights were measured individually every 14 d in the morning before feeding. From these values, we calculated the average body weights (BW) at different fattening stages, fattening durations, daily weight gain (DWG), daily concentrate feed intake (DCFI), and concentrate feed intake per 1 kg body weight gain (FCR).

### 2.3 Slaughter and carcass characteristics

At the target slaughter weights (Gr35, Gr40, and Gr45), six lambs from each group whose live weights were closest to the group mean were selected for slaughter (18 lambs in total). Lambs were fasted for 12 h before slaughter, with free access to water, and pre-slaughter live weights were recorded.

Immediately after slaughter, the weights of the skin, head, feet, testes, and visceral organs (heart, lungs including trachea and lobes, liver, gallbladder, and spleen) were recorded. Internal fat depots, including omental and mesenteric fat, were also weighed.

The gastrointestinal tract components, consisting of the esophagus, rumen–reticulum–omasum–abomasum (RROA), rumen, small intestine, and large intestine, were weighed first in the full state and then again after emptying and cleaning to determine their empty weights.

Hot-carcass weight was recorded immediately after slaughter, and the hot-dressing percentage was calculated. After chilling the carcasses at +4 °C for 24 h, cold-carcass weight, kidney weight, perinephric fat, pelvic fat, scrotal fat, and tail weight were determined, and cold-dressing percentage was calculated.

Empty-body weight was calculated by subtracting the weight of the gastrointestinal contents (the difference between the weights of the full and empty gastrointestinal tracts) from the pre-slaughter body weight. Subsequently, slaughter trait ratios were calculated relative to both pre-slaughter body weight and empty-body weight.

In order to determine the carcass characteristics, the carcasses were cut in half using a chainsaw at the level of the vertebrae. As a result of a modification of the method reported by Colomer-Rocher et al. (1987), the left side of the carcass was divided into seven joints: the hind leg, foreleg, back, loin, neck, breast, and flank. Physical dissection was performed to determine the percentages in each piece of lean, fat, bone, and losses (i.e., inedible parts). The ratios were calculated for each piece after weighing it. The Musculus longissimus dorsi (MLD) cross-sectional area (eye muscle area) of the carcass (between the 12th and 13th ribs) was drawn on parchment paper. Back fat depths were determined with a digital caliper on the same area from the side of the dorsal line. Carcass parts and dissection products were weighed with a 30 kg electronic scale sensitive to 5 g. The values obtained for the carcass parts were multiplied by 2 to obtain their whole-carcass weights.

### 2.4 Statistical analysis

All variables were calculated and presented as mean and standard error values. Parametric test assumptions, particularly conformity to normal distribution and homogeneity of group variances, were tested with the Shapiro–Wilk and Levene's Test, respectively. The variables that met parametric assumptions were compared using one-way ANOVA and Tukey multiple comparison test. The Bonferroni correction was applied after ANOVA to adjust p values for multiple comparisons. The MLD cross-sectional area in the carcass was calculated from the figure drawn on parchment paper using AutoDesk^®^ AutoCad 2022 software. A significance level of P < 0.05 was accepted for the statistical analyses, which were conducted using the SPSS 23.0 package.

## 3 Results

Table 2 presents the fattening performance data.

**Table 2:** Fattening performance of the lambs.

Items	Fattening groups (Mean ± SE)			P
	Gr35 ( n=12 )	Gr40 ( n=12 )	Gr45 ( n=12 )	
Final body weight (kg)	35.65 ± 0.99	40.28 ± 1.24	45.15 ± 1.10	
Fattening duration (day)	68.06 ± 3.47	92.76 ± 4.17	113.26 ± 4.69	
Average daily weight gain (kg)	241.50 ± 17.90	243.33 ± 29.68	235.45 ± 26.15	–
Daily concentrate feed intake (g d^−1^)	866.98 ± 123.11	963.10 ± 104.05	1034.10 ± 91.03	–
Feed conversion ratio^*^	3.590 ± 0.22	3.958 ± 0.28	4.392 ± 0.35	–

^*^ Concentrate feed consumed (kg) for 1 kg body weight gain. – P > 0.05.

Table 3 presents each group's mean slaughter weight characteristics. As a percentage of slaughter weights, the hot dressings for each group (Gr35, Gr40, and Gr45) were 46.22 ± 0.45 %, 45.49 ± 0.90 %, and 47.05 ± 0.83 %, respectively; the cold dressings were 45.26 ± 0.57 %, 44.66 ± 1.15 %, and 46.11 ± 0.85 %, respectively. As a percentage of empty-body weight, the hot dressings for each group were 54.01 ± 0.44 %, 53.01 ± 0.96 %, and 55.34 ± 0.28 %, respectively; the cold dressings were 52.90 ± 0.53 %, 52.05 ± 1.31 %, and 54.25 ± 0.96 %, respectively. Head percentages were 6.26 ± 0.14 %, 5.96 ± 0.24 %, and 6.15 ± 0.13 %, respectively. Skin percentages were 11.12 ± 0.53 %, 11.66 ± 0.33 %, and 12.12 ± 0.22 %, respectively. Digestive-tract-empty percentages were 7.72 ± 0.16 %, 7.63 ± 0.31 %, and 7.22 ± 0.15 %, respectively.

**Table 3:** Slaughter characteristics ( n=6 for each group).

Items	Slaughter weight groups (Mean ± SE)			P
	Gr35	Gr40	Gr45	
Slaughter weight (kg)	35.07 ± 0.16	40.80 ± 0.74	45.47 ± 0.49	
Empty-body weight (kg)	30.01 ± 0.29	34.99 ± 0.56	38.65 ± 0.32	
Hot-carcass weight (kg)	16.21 ± 0.14	18.52 ± 0.28	21.40 ± 0.48	
Cold-carcass weight (kg)	15.87 ± 0.16	18.19 ± 0.27	20.97 ± 0.46	
Hot dressing (%)	46.22 ± 0.45	45.49 ± 0.90	47.05 ± 0.83	–
Cold dressing (%)	45.26 ± 0.57	44.66 ± 1.15	46.11 ± 0.85	–
Hot dressing (%)^*#*^	54.01 ± 0.44	53.01 ± 0.96	55.34 ± 0.28	–
Cold dressing (%)^*#*^	52.90 ± 0.53	52.05 ± 1.31	54.25 ± 0.96	–
Non-carcass components (as percentage of slaughter weight)				
Head^*#*^	6.26 ± 0.14	5.96 ± 0.24	6.15 ± 0.13	–
Skin^*#*^	11.12 ± 0.53	11.66 ± 0.33	12.12 ± 0.22	–
Feet^*#*^	2.85 ± 0.02a	2.57 ± 0.07b	2.48 ± 0.05b	^***^
Heart (full)^*#*^	0.76 ± 0.04	0.72 ± 0.02	0.71 ± 0.02	–
Heart (empty)^*#*^	0.74 ± 0.03	0.70 ± 0.02	0.68 ± 0.02	–
Lungs^*#*^	1.82 ± 0.09a	1.63 ± 0.09ab	1.51 ± 0.06b	^*^
Trachea^*#*^	0.17 ± 0.007a	0.21 ± 0.004b	0.21 ± 0.012b	^**^
Lobs^*#*^	1.65 ± 0.08a	1.43 ± 0.09ab	1.31 ± 0.05b	^*^
Liver^*#*^	2.37 ± 0.07	2.29 ± 0.06	2.21 ± 0.07	–
Gallbladder^*#*^	0.09 ± 0.009	0.08 ± 0.019	0.08 ± 0.018	–
Spleen^*#*^	0.25 ± 0.016	0.27 ± 0.010	0.29 ± 0.016	–
Testicles^*#*^	0.49 ± 0.07a	0.69 ± 0.04ab	0.91 ± 0.07b	^**^
Omental fat^*#*^	0.95 ± 0.05a	1.06 ± 0.11ab	1.37 ± 0.12b	^*^
Mesenteric fat^*#*^	0.97 ± 0.05a	1.28 ± 0.09ab	1.41 ± 0.11b	^**^
Digestive tract (full)	21.03 ± 0.78	20.75 ± 0.31	21.12 ± 0.59	–
Esophagus	0.13 ± 0.007	0.13 ± 0.007	0.12 ± 0.005	–
RROA	12.62 ± 0.57	11.80 ± 0.36	12.53 ± 0.56	–
Rumen	10.83 ± 0.56	9.91 ± 0.21	10.57 ± 0.48	–
Small intestine	4.73 ± 0.29	4.85 ± 0.20	4.61 ± 0.18	–
Large intestine	3.55 ± 0.13	3.97 ± 0.13	3.86 ± 0.19	–
Digestive tract (empty)^*#*^	7.72 ± 0.16	7.63 ± 0.31	7.22 ± 0.15	–
Esophagus^*#*^	0.14 ± 0.009	0.15 ± 0.009	0.14 ± 0.007	–
RROA^*#*^	3.18 ± 0.14	3.24 ± 0.15	3.09 ± 0.08	–
Rumen^*#*^	2.29 ± 0.09a	1.97 ± 0.10b	1.89 ± 0.06b	^**^
Small intestine^*#*^	2.65 ± 0.08	2.55 ± 0.14	2.46 ± 0.06	–
Large intestine^*#*^	1.75 ± 0.04	1.68 ± 0.11	1.54 ± 0.08	–

– P > 0.05. ^*^ P < 0.05. ^**^ P < 0.01. ^***^ P < 0.001. ^a^, ^b^ Means with unlike letters in rows differ significantly ( P < 0.05). RROA: rumen–reticulum–omasum–abomasum. ^*#*^ Based on empty body weight.

Table 4 compares the carcass characteristics for the three groups. The lean percentages for each group (Gr35, Gr40, and Gr45) were 61.82 ± 0.60 %, 61.04 ± 0.47, and 57.53 ± 0.74, respectively ( P < 0.001). The fat percentages were 13.57 ± 0.24 %, 14.05 ± 0.17, and 19.46 ± 0.68, respectively ( P < 0.001). The bone percentages were 18.52 ± 0.68 %, 18.58 ± 0.42, and 17.99 ± 0.34, respectively ( P > 0.05). The tail fat percentages were 2.59 ± 0.37 %, 3.57 ± 0.39, and 3.42 ± 0.34, respectively ( P > 0.05). The leg percentages were 31.57 ± 0.71 %, 30.04 ± 0.58, and 28.41 ± 0.27, respectively ( P < 0.01). The foreleg percentages were 17.87 ± 0.28 %, 17.11 ± 0.37, and 17.72 ± 0.31, respectively ( P > 0.05). The back percentages were 13.39 ± 0.23 %, 12.63 ± 0.46, and 14.72 ± 0.56 %, respectively ( P < 0.05). The loin percentages were 8.41 ± 0.13, 7.88 ± 0.26, and 7.19 ± 0.12 %, respectively ( P < 0.001). The eye muscle areas were 14.29 ± 0.48, 16.16 ± 0.42, and 17.73 ± 0.20 cm2, respectively ( P < 0.001). The back fat depths were 1.13 ± 0.04, 1.57 ± 0.06, and 2.43 ± 0.28, respectively ( P < 0.001). The lean / fat ratio was highest in Gr35 and Gr40 (4.56 ± 0.10 and 4.35 ± 0.06) and lowest in Gr45 (2.98 ± 0.13) ( P < 0.001).

**Table 4:** Carcass characteristics ( n=6 for each group).

Items	Slaughter weight groups (Mean ± SE)			P
	Gr35	Gr40	Gr45	
Composition of the left half carcass (out of 100)				
Lean (%)	61.82 ± 0.60a	61.04 ± 0.47a	57.53 ± 0.74b	^***^
Fat (%)	13.57 ± 0.24a	14.05 ± 0.17a	19.46 ± 0.68b	^***^
Bone (%)	18.52 ± 0.68	18.58 ± 0.42	17.99 ± 0.34	–
Loses and uneatable parts (%)	1.04 ± 0.11	1.14 ± 0.09	0.95 ± 0.09	–
Tail fat (%)	2.59 ± 0.37	3.57 ± 0.39	3.42 ± 0.34	–
Kidneys (%)	0.75 ± 0.03a	0.67 ± 0.02b	0.65 ± 0.02b	^*^
Perinephric fat (%)	0.71 ± 0.08a	0.80 ± 0.07a	1.22 ± 0.15b	^**^
Pelvic fat (%)	0.43 ± 0.04	0.49 ± 0.04	0.47 ± 0.04	–
Scrotal fat (%)	0.43 ± 0.04	0.42 ± 0.04	0.54 ± 0.04	–
Lean / fat	4.56 ± 0.10a	4.35 ± 0.06a	2.98 ± 0.13b	^***^
Lean/bone	3.36 ± 0.12	3.29 ± 0.07	3.20 ± 0.06	–
Composition of the left half carcass cuts (out of 100)				
Leg	31.57 ± 0.71a	30.04 ± 0.58ab	28.41 ± 0.27b	^**^
Lean in leg	71.96 ± 0.29	72.36 ± 0.58	70.90 ± 2.55	–
Fat in leg	8.67 ± 0.29a	9.11 ± 0.44a	11.40 ± 0.25b	^***^
Bone in leg	19.38 ± 0.39a	18.53 ± 0.34ab	17.69 ± 0.35b	^*^
Loses and uneatable parts	0.49 ± 0.08	0.47 ± 0.06	0.48 ± 0.02	–
Foreleg	17.87 ± 0.28	17.11 ± 0.37	17.72 ± 0.31	–
Lean in foreleg	67.71 ± 1.08a	67.81 ± 0.63a	61.97 ± 0.63b	^***^
Fat in foreleg	10.98 ± 0.71a	11.34 ± 0.55a	18.98 ± 0.61b	^***^
Bone in foreleg	20.45 ± 0.61a	19.63 ± 0.23ab	18.41 ± 0.21b	^**^
Loses and uneatable parts	0.87 ± 0.24	1.22 ± 0.11	0.65 ± 0.06	–
Back	13.39 ± 0.23ab	12.63 ± 0.46a	14.72 ± 0.56b	^*^
Lean in back	54.94 ± 1.12a	58.88 ± 1.10b	53.78 ± 1.33a	^*^
Fat in back	18.62 ± 0.91a	14.08 ± 0.79b	20.75 ± 1.27a	^**^
Bone in back	25.05 ± 1.66	25.85 ± 0.90	24.53 ± 0.944	–
Loses and uneatable parts	1.39 ± 0.082	1.19 ± 0.17	0.95 ± 0.11	–
Loin	8.41 ± 0.13a	7.88 ± 0.26a	7.19 ± 0.12b	^***^
Lean in loin	64.47 ± 0.87a	62.61 ± 0.58ab	61.35 ± 0.95b	^*^
Fat in loin	16.90 ± 1.03	16.55 ± 0.67	18.44 ± 0.74	–
Bone in loin	17.59 ± 1.05	20.06 ± 1.14	19.04 ± 0.71	–
Loses and uneatable parts	1.05 ± 0.22	0.79 ± 0.23	1.16 ± 0.16	–
Neck	8.46 ± 0.56a	9.84 ± 0.29ab	10.61 ± 0.24b	^**^
Lean in neck	63.67 ± 1.64a	58.80 ± 0.73b	57.17 ± 0.99b	^**^
Fat in neck	12.17 ± 1.24a	15.69 ± 0.50b	14.56 ± 0.52b	^*^
Bone in neck	18.89 ± 0.33a	19.75 ± 0.53a	23.84 ± 1.14ab	^**^
Loses and uneatable parts	5.28 ± 0.45	5.77 ± 0.61	4.43 ± 0.71	–
Breast	9.50 ± 0.34	10.47 ± 0.21	9.99 ± 0.39	–
Lean in breast	52.64 ± 1.05	53.07 ± 0.45	46.07 ± 0.97	^***^
Fat in breast	23.29 ± 1.41	23.20 ± 0.94	32.16 ± 1.13	^***^
Bone in breast	24.07 ± 1.28	23.73 ± 0.75	21.78 ± 0.81	–
Flank	5.60 ± 0.40a	6.56 ± 0.51ab	7.17 ± 0.21b	^*^
Lean in flank	69.30 ± 0.98a	64.28 ± 2.02a	48.39 ± 1.99b	^***^
Fat in flank	30.70 ± 0.98a	35.72 ± 2.01a	51.61 ± 1.99b	^***^
Eye muscle area (cm^2^)	14.29 ± 0.48a	16.16 ± 0.42b	17.73 ± 0.20c	^***^
Back fat depth (mm)	1.13 ± 0.04a	1.57 ± 0.06ab	2.43 ± 0.28b	^***^

– P > 0.05. ^*^ P < 0.05. ^**^ P < 0.01. ^***^ P < 0.001. ^a^, ^b^, ^c^ Means with unlike letters in rows differ significantly ( P < 0.05).

## 4 Discussion

### 4.1 Fattening performance

Lalahan sheep contain approximately 75 % Kıvırcık and 25 % Akkaraman genotypes. In terms of fattening performance, Lalahan-genotype lambs are expected to be similar to the Kıvırcık breed. In this study, the DWG values obtained in fattening up to 35, 40, and 45 kg body weight (235, 242, and 236 g) were between the lower and upper limits of the DWG values (between 230–265 g) reported for intensively fattened Kıvırcık lambs (Akçapınar, 2000; Altın et al., 2005; Altınel et al., 1998; Demir et al., 2002; Erol, 2013; Gökdal et al., 2012). In a study conducted at Lalahan Livestock Research Institute, the DWG values of Kıvırcık × Akkaraman F2 and Kıvırcık × Akkaraman G1 lambs were lower than the DWG values for intensive fattening from 20 to 45 kg (271 and 279 g) and the values obtained for intensively fattened Bafra × Akkaraman F1 lambs from 20 to 34 kg (273 g) (Unal et al., 2006b; Yaranoğlu andÖzbeyaz, 2019).

The FCR values in the Gr45 group were higher than those in the Gr40 and Gr35 groups, but this difference was not statistically significant. That is, the lower daily feed consumption in the early stages of fattening is related to the lambs' smaller body weight and digestive system capacities. Regarding the FCR results for the three groups (3.590, 3.958, and 4.392 kg, respectively), between the beginning of fattening and slaughter weight, the results for the 35 and 40 kg groups were similar, while the 45 kg group lambs had the highest values. As expected, FCR increased with the increase in body weight during fattening, consistently with the literature (Yakan and Ünal, 2010; Yaranoğlu and Özbeyaz, 2019). On the other hand, the FCR values (3.590, 3.958, and 4.392 kg) obtained from the beginning of fattening to the 35, 40, and 45 kg body weights were lower than the FCR values (5.29–6.14 kg) reported for intensive fattening for Kıvırcık breed lambs (Altın et al., 2005; Demir et al., 2002; Gökdal et al., 2012). The values obtained for the Akkaraman × Kıvırcık crossbreed lambs were lower than those reported by studies with other lamb genotypes (Aytekin et al., 2015; Küçük et al., 2002; Yaranoğlu and Özbeyaz, 2019).

In terms of the DWG increase from intensive fattening, the Lalahan-genotype male lambs performed similarly to the Kıvırcık breed from which they originated but worse than Kıvırcık crosses and Akkaraman-breed lambs. On the other hand, in terms of FCR, they performed better than Kıvırcık and Kıvırcık crossbreeds, Akkaraman, and various local breeds. That is, they demonstrated high feed conversion ability, indicating that the Lalahan-genotype lambs show acceptable fattening performance under intensive conditions.

### 4.2 Slaughter and carcass characteristics

The slaughter characteristics were analyzed in relation to pre-slaughter and empty-body weights. The fullness of the digestive system may be at different levels in each animal, which may affect the differences between slaughter characteristics. Therefore, while evaluating the lambs' slaughter characteristics, the characteristics were also measured in relation to empty-body weight.

Both the hot- (46.22 %, 45.49 %, and 47.05 %) and cold-dressing (45.26 %, 44.66 %, and 46.11 %) percentages, calculated in relation to pre-slaughter body weight, were similar in the Gr35, Gr40, and Gr45 groups. Similarly, the hot- (54.01 %, 53.01 %, and 55.34 %) and cold-dressing (52.90 %, 52.05 %, and 54.25 %) percentages, calculated in relation to empty-body weight, were similar in the three groups. Although body weight at slaughter had no statistically significant effect on hot- and cold-dressing values, there was an increasing trend in yields with slaughter weight. This may be related to greater muscle and fat tissue as body weight increased. The hot-dressing values determined in our study were lower than those in Akkaraman, Bafra, and Bafra × Akkaraman F1 lambs, with an average slaughter weight of 34 kg (44.05 %, 44.61 %, and 46.77 %, respectively) (Yaranoğlu and Özbeyaz, 2019); Morkaraman and Kıvırcık × Morkaraman G1 lambs, with an average slaughter weight of 41 and 44 kg (46.15 % and 46.97 %, respectively) (Küçük et al., 2002); and Bafra lambs, with slaughter weights of 30, 35, 40, and 45 kg (44,19 %, 45.41 %, 47.16 % and 46.90 %, respectively) (Yakan and Ünal, 2010). Since slaughter yield is influenced not only by genotype but also by various factors related to production and management, these differences should be interpreted with caution. Differences in first-milk weaning weight, slaughter weight, fattening duration, feed energy and nutrient composition, gastrointestinal tract fill at slaughter, carcass fat deposition, and pre-slaughter handling can significantly affect slaughter yield. Therefore, the relatively lower slaughter yields observed in this study compared to some previous reports may be attributed not only to breed characteristics but also to differences in experimental conditions, and direct comparisons between studies should be interpreted accordingly.

In our study, across the three groups (Gr35, Gr40, and Gr45), feet ( P < 0.001) and rumen (empty) ( P < 0.01) ratios decreased, whereas omental ( P < 0.05) and mesenteric ( P < 0.01) fat ratios increased with increasing slaughter weight. The increase in omental and mesenteric fat ratios with the increase in slaughter weight was expected, consistently with previous studies (Yakan and Ünal, 2010; Yaranoğlu and Özbeyaz, 2019). The decrease in empty-rumen percentage is thought to be due to the inability of rumen development to keep up with the rate of muscle and fat increase, which is the main reason for the increase in body weight. The testicle ratio also increased with increasing slaughter weight ( P < 0.001), consistently with previous studies of local-breed lambs (Yakan and Ünal, 2010; Yaranoğlu and Özbeyaz, 2019). This can be explained by the approach of puberty due to the prolonged fattening period.

It is known that the weights of various parts of the carcass and the proportions of lean, fat, and bone in the carcass and carcass parts are directly related to slaughter weight and age. The increase in body weight is mostly due to the increase in muscle tissue in young animals and adipose tissue in adults, which affects the meat, bone, and fat ratios in the carcass and carcass parts (Akçapınar and Özbeyaz, 2021).

Increasing slaughter weight can affect carcass composition, specifically lower lean and bone percentages and higher fat percentages (Yakan and Ünal, 2010). In our study, the carcass lean percentages were similar in the Gr35 and Gr40 groups (61.82 % and 61.04 %, respectively) but lower in the Gr45 group (57.53 %); likewise, the carcass fat percentages were similar in the Gr35 and Gr40 groups (13.57 % and 14.05 %, respectively) but higher in the Gr45 group (19.46 %). The bone percentages were slightly lower in the Gr45 group (18.52 %, 18.58 %, and 17.99 %, respectively). It is noteworthy that there was a statistically insignificant increasing trend in the tail, pelvic, and scrotal fat percentages with increasing slaughter weight; however, the increase in perinephric fat percentages was significant ( P < 0.01).

The lean ratios for the Gr35, Gr40, and Gr45 carcasses (61.82 %, 61.04 %, and 57.53 %, respectively) were higher than many of those reported in native sheep breeds (Altınel et al., 1998; Yakan and Ünal, 2010; Yalcintan et al., 2017; Yılmaz et al., 2009). Given the high meat ratio in the carcass, the fat ratio in each group (13.57 %, 14.05 %, and 19.46 %, respectively) was lower than that reported for other domestic breeds. This is an important advantage of Lalahan lambs for lamb meat production.

The carcass bone ratios for each group (18.52 %, 18.58 %, and 17.99 %, respectively) were similar to those reported for native sheep breeds but lower than those for dairy sheep breeds (Unal et al., 2006a; Yakan and Ünal, 2010). This can be explained by the stronger skeletal structure of dairy sheep breeds compared to that of other sheep breeds. Compared to the Kıvırcık breed from which it originated, the Lalahan sheep genotype exhibits higher lean meat content, lower fat content, and similar bone ratios in the carcass. This has been considered to be desirable in terms of meat production. Furthermore, the Lalahan genotype is better than or similar to the local breeds in terms of carcass meat, fat, and bone ratios.

It is desirable that the proportion of prime-quality carcass parts (leg, back, and loin) in the carcass should be higher than the second- (foreleg) and third-quality (neck, breast, and flank) carcass parts. In our study, the ratios across the three groups for rump (31.57 %, 30.04 %, and 28.41 %, respectively), back (13.39 %, 12.63 %, and 14.72 %, respectively), and loin (8.41 %, 7.88 %, and 7.19 %, respectively) were similar to those of Kıvırcık lambs (Altınel et al., 1998; Yalcintan et al., 2017), noted for having good carcass quality, and Turkish Merino and Ramliç breeds (Yılmaz et al., 2009), noted for their meat-type traits. Therefore, the data obtained indicate that Lalahan lambs have suitable characteristics for meat production.

There is a relationship between the amount of quality lean in the carcass and MLD. MLD cross-sectional area (eye muscle area) is affected by factors such as genotype, fattening method, and body weight. In our study, eye muscle area increased significantly ( P < 0.001) with increasing slaughter weight. The eye muscle areas for the three groups (14.29, 16.16, and 17.73 cm^2^, respectively) were higher than those reported for Kıvırcık-breed lambs slaughtered at body weights of 40 (12.1 cm^2^) (Altınel et al., 1998), 32.14 (13.48 cm^2^) (Demir et al., 2002), 39 (15.26 cm^2^) (Gökdal et al., 2012), and 47.3 kg (14.24) (Yılmaz et al., 2009). Similarly, the eye muscle area values obtained in this study were found to be higher than most of the values reported for native sheep breeds raised to different slaughter weights and their crosses. This finding contrasts with those reported for Ivesi, Morkaraman, and Tuj (Macit et al., 2002); Akkaraman, Bafra, and Bafra × Akkaraman F1 (Yaranoğlu and Özbeyaz, 2019); and Akkaraman, Kıvırcık × Akkaraman, and Sakız × Akkaraman crosses (Unal et al., 2006a), as well as results reported for Bafra lambs evaluated at different slaughter weights (Yakan and Ünal, 2010). It is believed that this difference may stem not only from genotypic characteristics but also from variations in slaughter weight, fattening duration, feeding program, and rearing conditions.In our study, the back fat depth increased significantly ( P < 0.001) across the three groups (1.13, 1.57, and 2.43 mm, respectively). However, the fat depths were generally lower than those reported for native sheep breeds (Ünal et al., 2006b; Yılmaz et al., 2009). Overall, the slaughter and carcass characteristics of the Lalahan lambs in the present study can be considered to be satisfactory for meat production.

Some studies also show that the optimal slaughter weight in different sheep genotypes varies depending not only on carcass yield but also on fatness level and economic efficiency. While higher slaughter weights are preferred in meat-type genotypes in Europe, it has been reported that a live weight of approximately 38–42 kg provides the optimal balance between muscle development and fat deposition in medium-sized native or crossbred genotypes (Battagin et al., 2021; Prache et al., 2022). From this perspective, the optimal slaughter weight of approximately 40 kg determined for Lalahan lambs is consistent not only with the results of the present study but also with the physiological trends reported in the international literature.

The fact that the increase in the area of the Musculus longissimus dorsi (MLD) – a key indicator of muscle development – is more pronounced than the increase in back fat thickness is also a significant advantage of the Lalahan genotype in terms of meat production. Especially today, when consumer preferences are shifting toward lower-fat carcasses, the combination of high muscle development and limited fat deposition holds economic value for producers (Prache et al., 2022).

## 5 Conclusion

In conclusion, the Lalahan genotype exhibits balanced carcass composition, a high muscle-to-fat ratio, and controlled fat deposition not only when compared to the Kıvırcık but also when compared to the Akkaraman and other indigenous genotypes. These characteristics support the notion that this genotype is a viable alternative lamb suitable for intensive feeding in Türkiye's steppe ecology. The present study demonstrated that the Lalahan genotype is similar to or better than the Kıvırcık breed, its genotype originator, in terms of fattening performance, slaughter, and carcass characteristics. This indicates that the genotype has the potential to produce high-quality meat under steppe climatic conditions. Considering the favorable balance between growth performance and carcass composition, a slaughter weight of 40 kg can be recommended as the optimum slaughter weight for Lalahan lambs as it achieved the highest average daily gain while maintaining a high lean-meat percentage and a relatively low carcass fat percentage.

## Data Availability

All data can be obtained from the corresponding author.
